# Total coumarins of *Pileostegia tomentella* induces cell death in SCLC by reprogramming metabolic patterns, possibly through attenuating β-catenin/AMPK/SIRT1

**DOI:** 10.1186/s13020-022-00703-7

**Published:** 2023-01-03

**Authors:** Ying Liu, Kun Wu, Li Li, Fucui Zhu, Li Wang, Hua Su, Ying Li, Lu Lu, Guoshou Lu, Xiaoxi Hu

**Affiliations:** 1 Department of Pharmacology, Guangxi Institute of Chinese Medicine & Pharmaceutical Science, Nanning, 530001 Guangxi People’s Republic of China; 2 Guangxi Key Laboratory of Traditional Chinese Medicine Quality Standards, Guangxi Institute of Chinese Medicine & Pharmaceutical Science, Nanning, 530001 Guangxi People’s Republic of China; 3 Departments of Hepatobiliary and Gastrointestinal Surgery, Minzu Hospital of Guangxi Zhuang Autonomous Region, Nanning, 530021 Guangxi People’s Republic of China; 4 Department of Pharmacology, School of Pharmacy, Guangxi Medical University, Nanning, 530021 Guangxi People’s Republic of China; 5 Department of Pharmacy, Guangxi Orthopaedics and Traumatology Hospital, Nanning, 530012 Guangxi People’s Republic of China; 6 School of Medicine & Health, Guangxi Vocational & Technical Institute of Industry, Nanning, 530001 Guangxi People’s Republic of China; 7 Department of Chemistry, Guangxi Institute of Chinese Medicine & Pharmaceutical Science, Nanning, 530001 Guangxi People’s Republic of China

**Keywords:** Yao medicine, Pileostegia tomentella, Total coumarins, SCLC, Metabolism reprogramming

## Abstract

**Background:**

Small-cell lung cancer (SCLC) is a high malignant and high energy-consuming type of lung cancer. Total coumarins of *Pileostegia tomentella* (TCPT) from a traditional folk medicine of Yao minority, is a potential anti-cancer mixture against SCLC, but the pharmacological and molecular mechanism of TCPT remains largely unknown.

**Methods:**

Screening of viability inhibition of TCPT among 7 cell lines were conducted by using CCK-8 assays. Anti-proliferative activities of TCPT in SCLC were observed by using colony formation and flow cytometry assays. Morphological changes were observed by transmission electron microscope and Mito-Tracker staining. High Throughput RNA-seq analysis and bio-informatics analysis were applied to find potential targeted biological and signaling pathways affected by TCPT. The mRNA expression of DEGs and protein expression of signalling proteins and metabolic enzymes were verified by qPCR and Western blot assays. Activity of rate-limiting enzymes and metabolite level were detected by corresponding enzyme activity and metabolites kits. Xenograft nude mice model of SCLC was established to observe the in vivo inhibition, metabolism reprogramming and mechanism of TCPT.

**Results:**

TCPT treatment shows the best inhibition in SCLC cell line H1688 rather than other 5 lung cancer cell lines. Ultrastructural investigation indicates TCPT induces mitochondria damage such as cytoplasm shrinkage, ridges concentration and early sight of autolysosome, as well as decrease of membrane potential. Results of RNA-seq combined bio-informatics analysis find out changes of metabolism progression affected the most by TCPT in SCLC cells, and these changes might be regulated by β-catenin/AMPK/SIRT1 axis. TCPT might mainly decline the activity and expression of rate-limiting enzymes, OGDH, PDHE1, and LDHA/B to reprogram aerobic oxidation pattern, resulting in reduction of ATP production in SCLC cells. Xenograft nude mice model demonstrates TCPT could induce cell death and inhibit growth in vivo. Assimilate to the results of in vitro model, TCPT reprograms metabolism by decreasing the activity and expression of rate-limiting enzymes (OGDH, PDHE1, and LDHA/B), and attenuates the expression of β-catenin, p-β-catenin, AMPK and SIRT1 accordance with in vitro data.

**Conclusion:**

Our results demonstrated TCPT induces cell death of SCLC by reprograming metabolic patterns, possibly through attenuating master metabolic pathway axis β-catenin/AMPK/SIRT1.

**Supplementary Information:**

The online version contains supplementary material available at 10.1186/s13020-022-00703-7.

## Introduction

Small-cell lung cancer (SCLC) remains high malignant type of lung cancer, with over 70% of patients with SCLC presenting with disseminated lesions at diagnosis and suffering poor prognosis [1]. SCLC is a refractory lung cancer, for many decades, platinum compounds and topoisomerase inhibitors has long been the priority option for SCLC [2]. However, chemotherapy only shows partial remission in a short term and the patients quickly develop chemo-resistance within 1 year among most SCLC patients. Once the chemo-reagent fails to inhibit the tumor growth, relapse exacerbates SCLC progression and decreases survival possibility of patients [3]. In order to overcome the low respond of chemotherapy, many clinical trials raise the proposal with immune monoclonal antibody drug to seek good progress. Unfortunately, according to the records in clinical trials registration website (https://clinicaltrials.gov/), most of the proposals fail to be approved or even are suspended in the half way. Researchers still try to discover novel effective chemo-reagents for SCLC therapy from all aspects. New strategy in aspect of metabolism deprivation in tumor has been raised as potential targets, which might achieve good effects on SCLC [4–6].

SCLC is characteristics of fast proliferative ability and high metastatic tendency, which requires a large amount of energy consumption. For generating energy, SCLC cells motivate its metabolism including glycolysis, oxidative phosphorylation, pentose phosphate pathway and glutamine metabolism, to fully generate sufficient energy for proliferation [7, 8]. Recently, the expression of key metabolic enzyme or the activation of metabolic pathways has been reported as potential therapeutic targets in SCLC [6, 9, 10]. For instance, high serum lactate dehydrogenase (LDH) predicts recurrence or platinum resistance in SCLC patients with amrubicin therapy [10]. The high ratio of phosphoribosyl pyrophosphate amidotransferase (PPAT) to glutaminase (GLS1) enhances tumor growth including SCLC [6]. In addition, inhibition the site of PI3K/AKT/mTOR could down-regulate tumor progression through attenuating metabolism in SCLC [9].

Using of Chinese herbs extract has long history for Herbal medicines in traditional Chinese medicine. *Pileostegia tomentella* Hand. Mazz belongs to the saxifragaceae family, genus of *Pileostegia* Hook.f.et Thoms. In our local hospital, the boiled soup of *Pileostegia tomentella* has been widely used for unresectable refractory tumor which responded little to chemotherapy. However, the components showing antitumor effects were still uncovered. Our team devotes ourselves to material foundation, active fraction appraisal and pharmacological effects on *Pileostegia tomentella* Hand. Mazz. Firstly, total extract of *Pileostegia tomentella* (TEPT) showed proliferative inhibition in H_22_ tumor-bearing mice [11]. Based on this pharmacological effect, our chemistry team applied analytical methodology to set up standard quality control on total extract of *Pileostegia tomentella* [12]. Total coumarins of *Pileostegia tomentella* (TCPT), which took 56% proportion of TEPT, were major active fractions [13]*.* Recently, another local research team has found that TEPT induced canonical apoptosis pathway through over-producing reactive oxygen species in non-small cell lung cancer (NSCLC) [14]. TCPT is consisting of three coumarins components, skimmin (20%), 7-hydroxy-8-methoxycoumarin (10%) and umbelliferone (70%). Whether this mixture has anti-tumor effect on SCLC and what its mechanism of action will be explored and discussed in this study.

In the present study, first we treated 6 different types of lung cancer and 1 normal lung cell lines with series concentration of TCPT. Inhibition rate of TCPT treatment dropped the most in 2 SCLC cell lines, H446 and H1688. Morphological investigation indicated TCPT induced mitochondria damage in H1688 cells which might disturb metabolism pattern. Then RNA sequencing was introduced to systemically observe the changes caused by TCPT. Most of different expressed genes (DEGs) and the transcription of alternative splicing enriched in metabolic patterns in SCLC cells after treating cells with TCPT. Consistently, rescue assay applying si-RNA transfection and agonist indicated that the master metabolic pathway β-catenin/AMPK/SIRT1 was suppressed by TCPT treatment in SCLC. Additionally, TCPT treatment attenuated intracellular level of ATP and reduced the expression or activity of vital rate-limiting enzyme in aerobic oxidation progression. Moreover, in vivo model indicated that TCPT decreased proliferation and induced cell death in SCLC xenografts through Hematoxylin–Eosin (HE), Terminal Deoxynucleotidyl Transferase mediated dUTP Nick-End Labeling (TUNEL), and Ki-67 staining assays. The inhibition of β-catenin/AMPK/SIRT1 was also shown in SCLC xenografts as well as attenuation of expression or activity of rate-limiting enzyme in aerobic oxidation progression. Tumor tissues displayed drop of ATP level after administrating TCPT treatment. Based on these data, our study highlights TCPT induce cell death by reprogramming metabolic pattern, possibly through attenuating β-catenin/AMPK/SIRT1in SCLC.

## Methods

### Reagents and antibodies

The various antibodies used in this study are listed in Additional file 1: Table S1. According to our previous results, the major component of TCPT contained 7-hydroxycoumarin (70%), skimmin (20%) and 7-hydroxy-8-methoxycoumarin (10%). The details of each chemical are listed in Additional file 1: Table S1.

### Cell culture

Cell lines H1299, A549, H1688, H446, H226 were purchased from Shanghai Cell Bank, National Collection of Authenticated Cell Cultures, cell lines BEAS-2B and SK-MES-1 were purchased from Kunming Cell Bank, National Collection of Authenticated Cell Cultures. The culture conditions of each cell lines were applied as described in Additional file 2: Table S2.

### CCK-8 assay

A series of TCPT medium (12.5, 25, 50, 100, 200 μg/mL) was prepared by half dilution method, each cell lines were set as 8 types of groups, background (no cells, medium only), blank, 5 TCPT treatment. Different lung cancer cells and normal lung bronchiole alveolar cells BEAS-2B were harvested and seeded in each well of 96-well plate of blank and TCPT treatment group, each well containing 5 × 10^3^ cells. Seeded cells were cultivated in another 24 h, and then culture mediums were replaced by 100μL normal or TCPT medium. All cells were allowed to grow another 48 h under the condition with or without TCPT medium. At the end of the TCPT treatment, 10μL of CCK-8 reagent was added into each well of 8 groups. CCK-8 reaction time varied from 1 to 3 h according to preliminary experiment of each cell lines. The microplate reader (SynergyH, BioTek, USA) was used to detect OD value of each well at the wavelength of 450 nm. Cell viability (%) was calculated according to formula: (OD_TCPT_-OD_background_)/(OD_blank_-OD_background_) × 100%, Inhibition (%) = 100%—Cell viability (%).

### Plate colony formation assay

H1688 cells were harvested and re-suspended in a density of 5 × 10^3^ cells/mL, and then a volume of 100μL cells suspension was seeded in each well of a 6 well plate. After 24 h incubation, medium of each well was replaced by 2 mL normal medium, or different TCPT medium (25 μg/mL, 50 μg/mL, or 100 μg/mL).Cultivation lasted 10 days. At the endpoint, medium of each wells were discarded, then cells were washed by PBS and fixed with cold ethanol for 10 min, followed by staining with 0.1% crystal violet for 10 min. After staining, cells were washed by PBS twice and then were observed under the FluorChem R (Protein simple, US) with colony formation scanning model.

### Flow cytometry (FCM) for detecting cell death and viability

A total of 5 × 10^5^ H1688 cells were implanted in each well of 6 well plates. Twelve hours later, medium of each wells were changed by new blank medium or conditioned TCPT medium (25 μg/mL, 50 μg/mL, or 100 μg/mL) according to group setting. TCPT treatment lasted 48 h, cells of each well were harvested by 0.25% trypsin and then cells were centrifuged with 800 g 10 min at 4 °C. A volume of 500μL binding buffer was added to resuspend cells followed by addition of 5μL Annexin V-FITC dye and 5μL PI dye for staining. Detection of stained cells suspension were conducted by using GUAVA Flow Cytometer (easyCyte HT, Merck Millipore, US).

### Transmission electron microscope (TEM)

A total of 1 × 10^6^ H1688 cells were cultured in each T25 cell culture flask and was treatment with normal medium (control group) or 100 μg/mL TCPT (TCPT group) for 24 h respectively. Cells of each flask were collected and fixed with 2.5% glutaraldehyde 18 h at 4 °C. Then cell pellets were washed by cold PBS three times, followed by being post-fixed with 1% OsO_4_ for 2 h at 25 °C. Fixed cells were dehydrated with graded ethonal-actone solution (50% acetone, 70% acetone, 80% acetone, 90% acetone and pure acetone) and embedded in epoxy resin. After cutting blocks into 70 nm slides, uranyl acetate and lead citrate were used to stain cell slides. Cell slides of each group were observed by TEM (H-7650, HITACHI, Japan).

### Mitochondrial fluorescent tracer

H1688 cells were seeded in 12-well plate containing sterile glass coverslips with density of 1 × 10^5^/well. The other day, culture medium of each wells were replaced by 1 mL normal medium or 100 μg/mL TCPT conditioned medium. Well plates were placed in incubator with 5% CO_2_ at 37 °C for another 24 h. MitoTracker (cat. C1049B, Beyotime, Shanghai) were diluted with FBS-free medium in a ratio of 1:1000 to form staining working solution. The culture medium in each well were discarded and changed to 1 mL working solution for 1 h staining at 37 °C. Nuclear was stained with DAPI (10 μg/mL), and then cover slips then were sealed with glycerinum. Fluorescence microscope (Evos FL Auto, Life, USA) was applied to capture the mitochondria state in live SCLC cells. Fluorescence intensity presented mitochondrial potential and was quantitated by ImageJ.

### High Throughput RNA-seq analysis for screening potential targets of biological function and signalling pathways after TCPT treatment

Petri dishes (diameter 10 cm) were used to grow H1688 cells. When the confluence of cultured cells reached 90%, medium of each dishes were replaced by blank medium or 100 μg/mL TCPT medium, and cells were incubated for another 48 h. RNA extraction was proceed by adding 1 mL RNAiso plus (TAKARA, Japan) in each dishes of both blank group and TCPT treatment group, respectively. Each group set triplicates at the same time. At least 100 μg of total amount of RNA were used to establish cDNA library. High throughput screening was conducted by Shanghai Applied Protein Technology (Shanghai, China). Briefly, mRNA in each samples were enriched by magnetic bead with Oligo (dT), and then double strain cDNA were formed by DNA polymerase I and purified by AMPure XP beads, respectively. The purified cDNA library of each sample then was pooled to the same molecular concentration. Sequencing was performed by using Illumina NovaSeq 6000 system (San Diego, CA). Read counts were analysed by DESeq2: a |log_2_foldchange|> 1 and corrected P value (padj) < 0.05 were considered as the threshold of differentially expressed genes. Bio-informatics analysis for Gene ontology and KEGG enrichment were performed by using Fisher’s Exact Test, and then visualized to bar charts of GO terms and KEGG mappings. Database of STRING, AnimalTFDB/PlantTFDB or Pfam and rMATS were introduced to analyse protein–protein interaction and alternative splicing.

### RNA extraction and quantitative reverse transcription-polymerase chain reaction (RT-qPCR) assay

H1688 cells were seeded in each well of a 6-well plate, with a density of 5 × 10^5^/mL. After 24 h incubation, medium in each plates were replaced by normal medium, 50 μg/mL TCPT medium or 100 μg/mL TCPT medium. H1688 cells treated with or without TCPT were collected and lysed by RNAiso plus (TAKARA, Japan) after 48 h incubation, respectively. RNA were extracted by chloroform, isopropanol and purified by cold 75% ethanol. Then Nanodrop One (Thermo Fisher, US) was used for detecting the concentration and purity of total RNA. One microgram total RNA of each samples were mixed with reverse transcription reagent and were reacted into cDNA following the manufacturer instruction. The reset of RNA samples were stored in −80 °C.

RT-qPCR was performed according to the manufacturer instruction. A total amount of 100 ng cDNA templates were loaded into SYBR Green mixture to form a 20 μL volume of reaction system, and then reactions were processed by thermal cycler (LightCycler 480 II, Roche, USA). All the primers used are listed in Additional file 3: Table S3.

### Transient transfection of siRNA

H1688 cells were seeded in each well of 6-well plate with a density of 5 × 10^5^ per well. A total of 5 μM si-RNA was mixed with 10 μL Lipo2000 reagent in 250 μL basic medium to form transfected mixture (lipidosome of siRNA). Before adding lipidosome of siRNA, then cultured medium of each well was replaced by 2 mL basic medium. The formation of lipidosome of siRNA was processed at 25 °C for 30 min following the manufacturer instruction. And then the mixture was added into each well of 6-well plate. Incubation of transient transfection was set for 6–8 h followed by routine replacement of complete medium of each well daily.

### Western blot assay

A total of 5 × 10^5^ H1688 cells were seeded in each well of 6-well plate. Medium of each wells were replaced by blank medium or TCPT medium (50 μg/mL, 100 μg/mL) for cultivating another 48 h. Cells of each well were collected and lysed with RIPA buffer, followed by protein extraction using centrifuge with 12000*g*, 15 min, at 4 °C.

About 20 mg tumor tissues of each xenograft individuals were collected into 2 mL centrifuge pipe which are containing 200μL RIPA buffer. Protein extractions of tissues were conducted by using FastPrep-24^Tm^ 5G Grinding machine (S matrix model, MP Biomedicals LLC, US) with 30 s, at 25 °C. After centrifuging tissue blending mixture with 12000*g*, 15 min, at 4 °C, protein supernatant was translocated in a new pipe for western blot assay.

Protein concentration of cells and tissues sample were quantified by using BCA assay kit. Loading sample containing 20 μg total protein was added in each lines of SDS-PAGE gel for electrophoresis with 100 V, 2 h. PVDF-membrane (0.22 μm) then was used for transferring separated protein from gel to membrane. Then membrane was blocking with 5% non-fatty milk. Blocked membrane then was incubated with various diluted primary antibody solution (Additional file 1: Table S1) overnight at 4 °C for immunobloting. Diluted secondary antibody then was added and incubated 2 h at 25 °C, after washing membrane three times with PBST solution. Electrochemiluminescence solution (Vazyme E412, Nanjing) was prepared and used for developing image of protein band. Tannon 4600 imaging system (Tannon, Shanghai) was applied to capture specific protein band and further analysis.

### Activity of metabolic enzyme and content of metabolite assays

Preparation of cells with different TCPT treatment.

Cells: Cells were harvested and adjusted with density of 1 × 10^6^ /mL, and a volume of 500μL cells suspension was added in each well of 6-well plate. After 12 h incubation, culture medium of each wells were changed by new blank medium or TCPT medium (50 μg/mL or 100 μg/mL) for another 48 h.

Tissues: A weight of 20–50 mg xenograft tumor tissues in each groups were grinded with 200 μL–500 μL extracting solution of assay kit. Different assay kits required specific extracting solution according to manufacturer instruction.

#### Assays of glucose level

Cells of each well were collected and lysed by 20% NP-40 solution. About 50 mg tumor tissue of each groups were mixed and grinded with 20% NP-40solution, then the supernatant was collected and temporarily stored at 4 °C before detection. A volume of 10 μL cell or tissue lysis was added into 180 μL glucose detecting working solution. This reacting mixture was heated at 95 °C for 8 min then cooled at 4 °C for standby. A total of 180 μL mixture was transferred to a 96 well plate, and the OD value of each well was read at 630 nm wavelength of microplate reader. OD value was then introduced into standard curve to calculate the glucose level for each sample.

#### Assays of adenosine triphosphate (ATP) level

Cells of each well or 20 mg tumor tissue were lysed with extracting solution. Cell or tissues supernatant were separated by centrifuge at 4 °C. Detecting working solution was prepared followed by the manual instruction. Transferring 100μL working solution to a 96 well white plate and pipetting 20 μL of each samples of cell or tissue supernatant. Luminous signal value was recorded by microplate reader, integral time of each well was 10 s. Luminous value was then introduced into standard curve to calculate the ATP concentration for each sample.

#### Assays of lactate (LA) level

At the endpoint of treatment, cell supernatant and cells of each well were collected separately and pretreated with LA extracting solution. Approximately 30 mg of tumor tissue in each group were minced and pretreated with LA extracting solution. Sample solution and then were reacted with detecting kit at 37 °C for 20 min to precipitate methyl thiazolyltetrazolium. LA in each sample would be reacted and generated to methyl thiazolyltetrazolium. Ethanol was used to resolve precipitation and microplate reader was applied to record the OD value at 570 nm wavelength. OD value was then introduced into standard curve to calculate the LA level for each sample.

#### Assays of LDH activity

The extracting supernatant of cells in each well and tumor tissue homogenate of each group were collected for detection. Detecting reagents were mixed to generate testing tube and reference tube for each sample. A volume of 50 μL sample solution was added into testing tube and reference tube respectively and incubated at 37 °C for 30 min. The generation of pyruvate dinitrobenzene hydrazine presented the activity level of LDH of each sample. The microplate reader was applied to record the OD value of pyruvate dinitrobenzene hydrazine solution at 450 nm wavelength. OD value was then introduced into standard curve to calculate the LDH activity for each sample.

#### Assays of HK activity

Working solution was prepared ahead of detection, according to the manual instruction. Cells of each well or 20 mg tumor tissues were lysed with extracting solution by ultrasonication. After 8000*g* centrifuging at 4 °C for 10 min, 10 μL of each sample supernatant was mixed with 190 μL of working solution. The generating amount of NADPH presented the activity level of HK of each sample. Then, the testing solution was read at 340 nm by microplate reader at the time point of 20 s (A1) and 5 min 20 s (A2), separately. The HK activity unit of each sample was calculated as K × (A2-A1).

#### Assays of PDH activity

Cells and tumor tissues (50 mg) were lysed with extracting solution by ultrasonication. Testing supernatant of each sample was collected by centrifuge. Working solution was prepared and pre-incubated at 37 °C before extracting samples. A volume of 10 μL sample supernatant or diluted water was added into 180 μL of working solution respectively and incubated at 37 °C for 10 min. The decrease of 2, 6-dichlorophenol in dophenol presented the PDH activity of each sample. Microplate reader should be preheated at 37 °C for 30 min before detection, the OD value of testing solution or blank solution of each sample was read at 605 nm at 10 s(A1) and at 1min10s(A2) respectively. ΔA_testing_ or ΔA_blank_ = A1-A2, The PDH activity unit of each sample was calculated as K × (ΔA_testing_-ΔA_blank_).

### Subcutaneous xenograft tumor in nude mice

The 4-week-old nude mice (weighed 14–16 g) were enrolled in this study. Mice were randomly divided into three groups: solvent, TCPT treatment (low dose and high dose). Each group consisted of half males and half females. Before implanting xenograft tumor cells, all mice were anaesthetised by intraperitoneal injection of urethane (0.5 g/kg). We then performed a subcutaneous injection with a 100 μL H1688 cells (5 × 10^6^ cells/mL) at the axilla of fore limb. Nude mice were housed in a specific pathogen-free (SPF) room at the experimental animal centre of Guangxi Institute of Chinese Medicine. After 5 d, mice in TCPT treatment groups received intraperitoneal injections of TCPT (50 mg/kg, or 100 mg/kg, every 2 days) while solvent group were received 100 μL solvent for 20 days. The tumor volume was measured by verniercaliper every 2 days so did recording the body weight of each mouse. At the end of the observation, mice were sacrificed and their major organs were resected for evaluating toxicity. Xenograft tumors were resected and weighed. A part of each tumor was cut into pieces for protein extracting or activity of metabolic enzyme and content of metabolite assays, half of tumor of each groups were paraffin embedding. The rest of tumor tissues were stored at − 80 °C for further use. Tumor volumes were calculated by the equation: ‘V = (Long diameter × Short diameter^2^)/2’ [15].

All animal procedures were performed in accordance with the guidelines for the ethical review of animal welfare (GB/T35892-2018). This study was approved by Animal Ethics Committee of Guangxi Institute of Chinese Medicine (NO. 20220517016).

### Hematoxylin–eosin (HE) staining

Tissue slides from the tumor xenograft were de-paraffinised after warming in oven at 70 °C for 1 h. Then, by stepwise soaking the slides in four jars of xylene, each jar was left for 5 min. All slides were rehydrated with pure ethanol, 90% ethanol, 70% ethanol, 50% ethanol, and dilution water, each jars was left for 5 min. Firstly, rehydrated slides was soaked in haematoxylin for nuclear staining for 5 min, followed by eosin staining of the cytoplasm for 10 min. Then all slides were quickly rinsed with 1% hydrochloric acid of ethanol for 5 s. The coverslips were sealed with permount TM mounting medium in each slider after ovening 1 h at 50 °C. The staining results were then observed and recorded using an optical microscope at 200× magnification.

### TUNEL assays

The deparaffinage and rehydration procedure were conducted as the description in (2.11 HE staining). Rehydrated tissue slides were digested with protease K (20 μg/mL) at 25 °C for 15 min. TUNEL probe was prepared by mixing TDT enzyme and fluorescence labelling solution together. After being washed with PBS, each slide of tumor tissues were incubated with 50 μL TUNEL probe at 37 °C for 1 h. DAPI (10 μg/mL) solution was used to stain cell nucleus, followed that all slides were sealed with glycerine. TUNEL staining of all slides were observed by using fluorescence microscope (Leica LAS X) at 200× magnification.

### Immunofluorescence (IF) assays

The deparaffinage and rehydration procedure were conducted as the description in (2.11 HE staining). Heat induced epitope retrieval was introduced to all rehydrated slides with citrate buffer using microwave oven for 10 min. Slides were blocked with 5% goat serum solution for 1 h at 25 °C. The primary antibodies of Ki-67 (Additional file 1: Table S1) were dropped in tissue slides and then were placed overnight at 4 °C for incubation. Then, tissue slides were incubated with diluted fluorescent secondary antibody solution (Additional file 1: Table S1) at 37 °C for 1 h. Nuclei staining were performed by using DAPI staining at 25 °C for 5 min. When staining was done, coverslip was sealed with glycerol in slider. Staining results were recorded by taking picture of optical microscope at 200× magnification. The average of density of fluorescent signal of each slide was record by using ImageJ.

### Statistical analysis

Experiments were repeated at least three times independently, and at least triplicates were set by each independent time. The statistical results of each figure are indicated as the mean value ± SD. SPSS 19.0 software was applied to process date with statistical analysis. Statistical significance was determined using the Student’s unpaired two-tailed t-test or one-way ANOVA multiple comparison test, enumeration data was analysed by chi-square test, as indicated in the legend (*P < 0.05, and **P < 0.01).

## Results

### TCPT induces cell death in SCLC cells

**Fig. 1:**
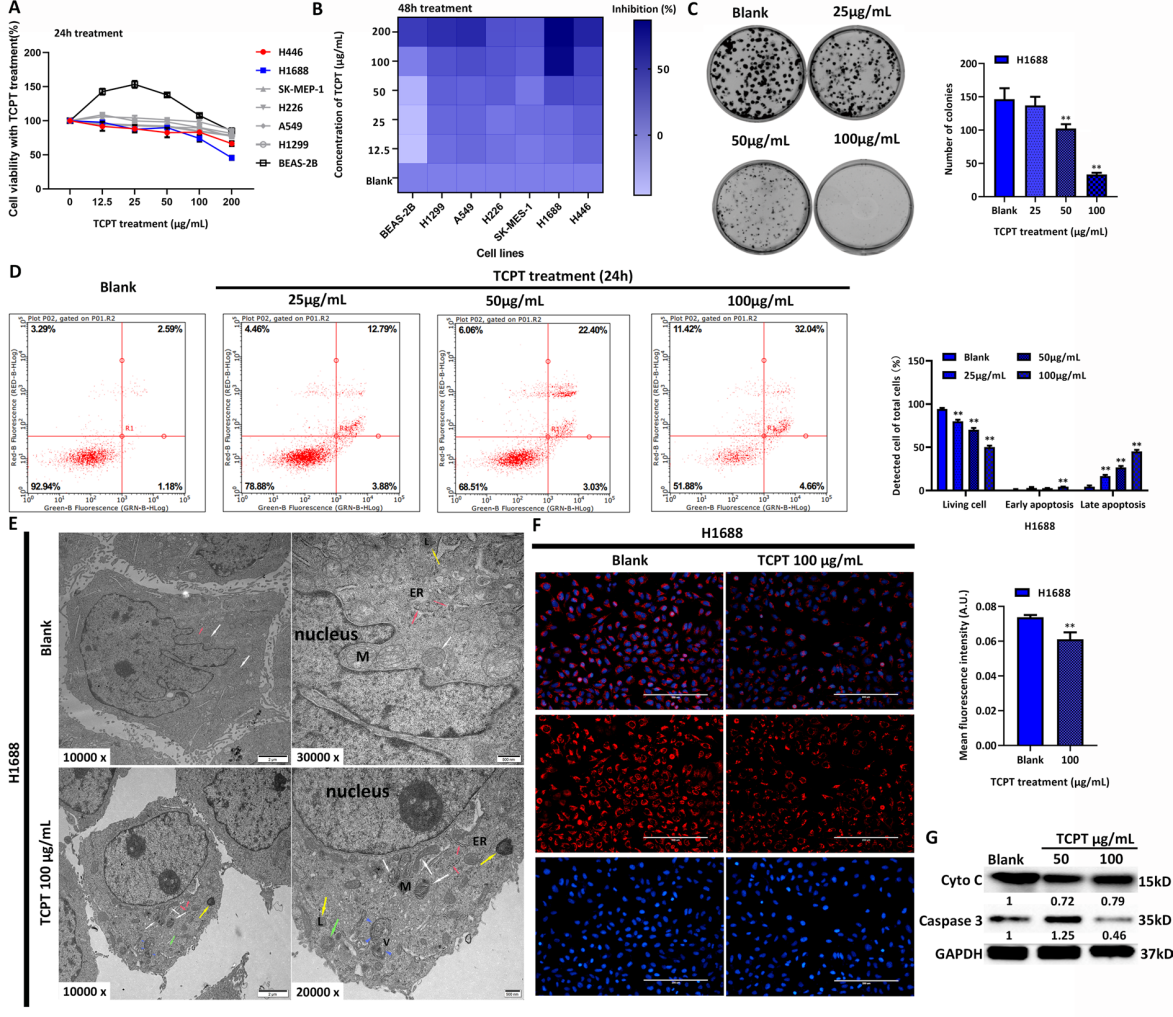
TCPT induces cell death of SCLC. **A** cell viability of various lung cancer cell lines and normal bronchial epithelium lung cell lines BEAS-2B (black label) after treating TCPT in 24 h. **B** Inhibition rate of 48 h-treatment of TCPT with different concentration among above cell lines. The deeper the Block color, the higher the inhibition rate of TCPT treatment. **C** Colony formation results of H1688 cells when treating TCPT. Left panel indicates the colony numbers under each concentration of TCPT, right panel displays the count numbers with statistic analysis. **D** FCM detects cell viability and death in H1688 cells when treating with series concentration of TCPT in 24 h. Bar chart in right panel shows percentage of cell in specific quadrant. Lower right quadrant presents single positive of FITC-Annexin-V staining, which is a classical feature of early apoptosis, thereby presenting in bar chart as early apoptosis. Upper left quadrant (single positive PI staining) and upper right quadrant (double positive FITC-Annexin-V/PI) presents late apoptosis cells, since the ferroptosis, pyroptosis, programmed necrosis or other death type shares the similar feature as late apoptosis. **E** observation of cellular ultrastructure with TEM among H1688 with TCPT treatment and its blank counterpart. White arrows indicate mitochondria, pink arrows indicate endoplasmic reticulum, yellow arrows indicate lysosomes and blue arrowhead indicate vesicas encapsulated other organelles. **F** staining of mitochondria using Mito-Tracker probe. Decreased amount of intracellular dye indicates the attenuation of mitochondrial membrane potential. Bar chart presents the semi-quantitative analysis of mitochondrial staining. **G** protein expression of cytochrome C, caspase 3 and claved-caspase 3 among H1688 with different TCPT treatment and its blank counterpart. *, ** vs blank group, present P value < 0.05 and 0.01 respectively

We previously found TCPT showed anti-proliferative activity in colorectal cancer cell lines and breast cancer cell lines [13], but no data was indicated in lung cancer. Therefore, TCPT was firstly applied to proliferation screening among lung cancer cell lines. Six lung cancer cell lines and 1 normal lung bronchial epithelial cells BEAS-2B were treatment with series of concentration of TCPT. In Fig. 1A, B, inhibition of the proliferation of BEAS-2B is observed at high dose of TCPT, starting from 100 μg/mL, while the TCPT inhibited the viability of other lung cancer cells in a dose dependent manner within 24 h (Fig. 1A) and 48 h (Fig. 1B), starting at low dose of TCPT (< 50 μg/mL). Among 6 lung cancer cell lines, the inhibition rate of small cell lung cancer (SCLC) H1688 showed the sharpest downtrend when treating with TCPT in 24 h and 48 h. Then Fig. 1C illustrated that colony formation of H1688 cells was decreased while the concentration of TCPT increased in a long-term period. FACS result in Fig. 1D showed that TCPT treatment significantly attenuated the proportion of living cells, in the meantime dramatically raised late apoptosis rate but slightly induced early apoptosis in H1688 cells. Since the characteristic of late apoptosis was caused by other cell death pathway, next transmission electron microscope was applied to observe the ultrastructure of H1688 cells after TCPT treatment (100 μg/mL). In Fig. 1E, TCPT treatment induced the pathological change of mitochondrial such as mitochondrial cytoplasm shrinkage, mitochondrial ridges concentration and early sight of autolysosome. Besides, more high-density lysosomes and big phagocytic vesicle appeared which might imply degradation was ongoing among proteins and damage organelles. In addition, 100 μg/mL TCPT could also impair permeability of mitochondrial membrane and decreased mitochondrial potential which was observed by Mito-Tracker staining (Fig. 1F). Besides, the protein expression of cytochrome C shows decreased when treating TCPT in H1688 (Fig. 1G). However, the expression of caspase 3 shows a slight decrease in H1688 cells with 100 μg/mL TCPT while an mild increase of caspase 3 expression was detected in H1688 cells with 50 μg/mL TCPT, compared with the blank counterpart (Fig. 1G). In summary, these results suggest that TCPT could induce cell death in H1688 cells through damaging mitochondria.

### The potential biological and signalling pathways affected by TCPT were screened out by the next generation sequencing RNA-seq in SCLC

**Fig. 2:**
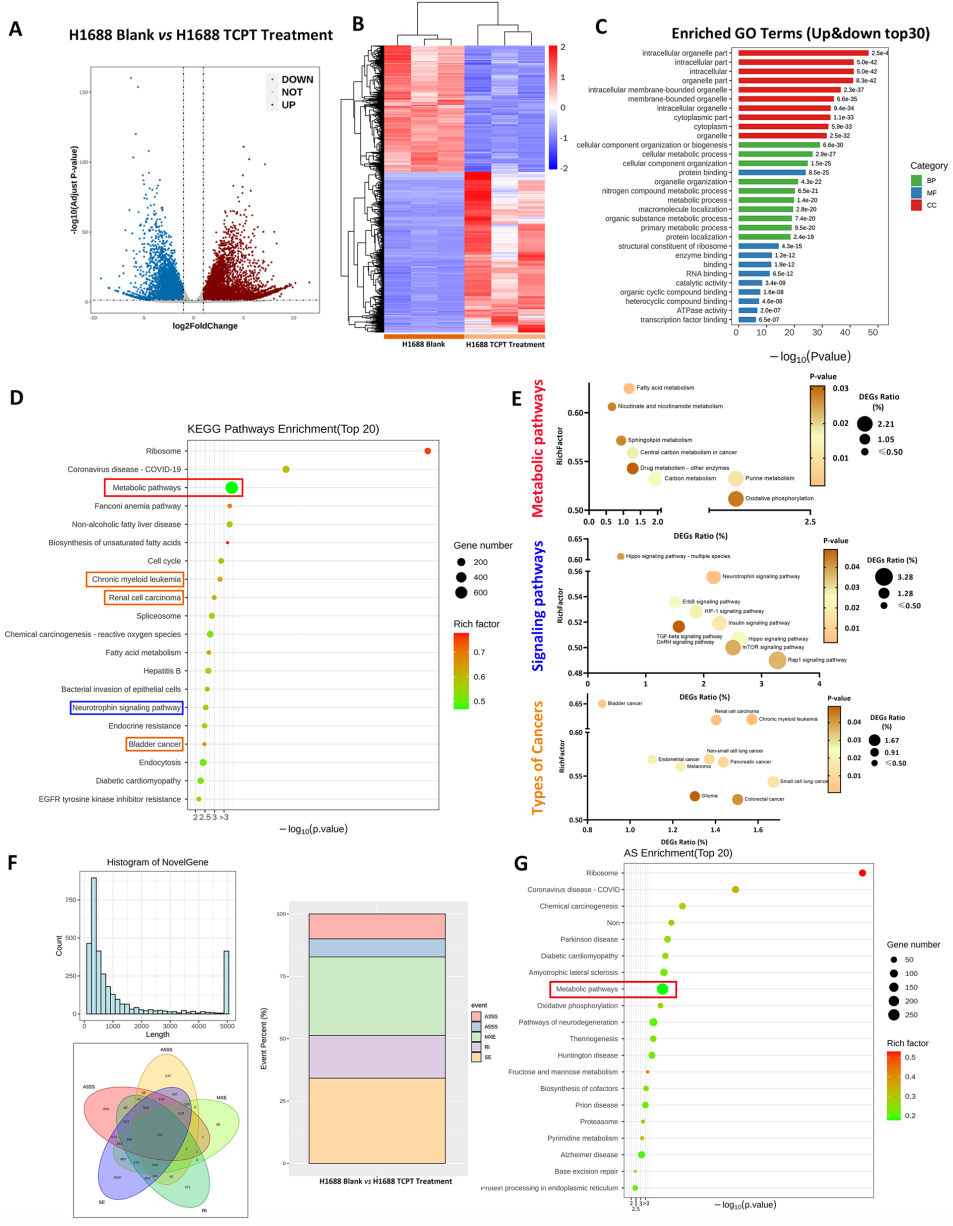
bio-informatics results of high-througput RNA-seq analysis among H1688 cells with TCPT and their blank counterpart. **A** Volcano plot of DEGs between H1688 TCPT treatment group and its blank counterpart. The threshold of DEGs sets up as padj (corrected p value) < 0.05 and ∣log_2_foldchange∣ > 1. Down-regulated DEGs present as blue dots while up-regulated DEGs present as red dots. **B** Heatmap of of DEGs between H1688 blank *vs* H1688 TCPT treatment. Red block presents up-regulated DGEs, whereas blue block presents down-regulated DGEs. Upper and left dendrogram indicate the relationship of expression pattern and amount in individual samples, respectively. **C** Bar chart of total enriched GO terms of DEGs (rank top 30). The color of columns present category of biological process (BP), molecular function (MF) and cell component (CC), respectively. Ordinate shows the GO terms, and abscissa shows p-value of enrichment analysis. **D** Bubble diagram of KEGG enrichments of DEGs (Top 20). Ordinate shows the GO terms, and abscissa shows p-value of enrichment analysis. The size of bubble shows the enriched DEGs number, and the color of bubble presents the rich factor of each terms. Boxed terms are the representative of metabolism pathways (red box), signaling pathways(blue box) and types of cancer(orange box), respectively. **E** Classification of all terms to the category of metabolism pathways, signaling pathways or types of cancer. Ordinate shows the rich factor of each terms, abscissa and the size of bubble present proportion of subpsrietal DEGs of total DEGs, and color of size presents p-value of each terms. **F** Summary of novel genes and alternative splicing. Histogram of novel gene shows the length and number of found gene that cannot be referenced to library upper left panel). Venn chart indicates the types of alternative splicing (AS) among DEGs (lower left panel) and the event percentage of each type of total events(right panel). A3SS presents alternative 3’ splice site, A5SS presents alternative 5’ splice site, MEX presents mutually exclusive exons, RI presents retained intron and SE presents skipped exon. **G** KEGG Enrichment of AS. Ordinate shows the pathway terms, and abscissa shows p-value of enrichment analysis. The size of bubble shows the enriched DEGs number, and the color of bubble presents the rich factor of each terms. Red rectangle boxed the most DEGs enriched term-metabolic pathway

Later we tried to find out how TCPT systematically affected SCLC cells. Normal H1688 cells and their TCPT treated counterpart were introduced to mRNA next generation sequencing. The volcano graph and heatmap chart in Fig. 2A, B illustrated a total of 11,429 differentially expressed genes (DEGs) were screened out and clustered, wherein 6397 of them were up-regulated and 5032 of them were down-regulated. Table 1 listed top 30 up-regulated and down-regulated DEGs and randomly selected DEGs (for validation), providing the targets verification for our further research. Since TCPT contains three components, it is more rational focusing on the whole biological process and pathway enrichment, rather than picking some special targets. Figure 2C displayed DEGs enriched in the gene ontology terms of intracellular organelle of cell component, cellular metabolic process of biological process and protein binding of molecular function. KEGG pathways enrichment (Fig. 2D) indicated the most DEGs belonged to metabolic pathways such as oxidative phosphorylation, followed by three types of cancer (chronic myeloid leukemia, renal cell carcinoma and bladder cancer) (Fig. 2E, top panel). Besides, 9 signalling pathways were found out that might predominately respond to TCPT treatment (Fig. 2D, E, middle panel)), which provided us potential orientation to further discover molecular mechanism. Additionally, DEGs also related to 10 types of cancer (Fig. 2D, E, down panel), which might be the hints that TCPT might have anti-tumor effects in these cancer. Surprisingly, there were 2190 novel genes (Fig. 2F) were identified. The major proportion of them either had short length, below 500 bp or longer length, above 5000 bp. Short novel gene might generate new small molecular protein or new lncRNA regulator. Furthermore, TCPT treatment induced alternative splicing of genome in SCLC, wherein over 50% of events were exon skipping and mutually exclusive exons (Fig. 2F, right panel). Events of alternative splicing of genome might be one reason that generated novel genes. Next we found out that most DEGs which caused events of alternative splicing enriched in metabolic pathways (Fig. 2G).

**Table 1:** List of up-regulation and down-regulation of DEGs

	Up-regulation DEGs			Down-regulation DEGs			
No	Symbol	Log_2_FC	q-value	No	Symbol	Log_2_FC	q-value
1	HSPA6	8.3212	1.70 E−42	1	CEACAM7	− 9.225	1.63E−17
2	Metazoa_SRP	7.9155	2.83E−08	2	TSTD2	− 8.3844	1.18E−10
3	RELN	7.4819	0.11965	3	UGT2A3	− 7.684	1.22E−08
4	ARC	7.2434	2.48E−24	4	ACTBP2	− 7.598	1.89E−08
5	GADD45G	7.0926	5.90E−06	5	SRXN1	− 7.4165	5.30E−11
6	TNNI3	6.9963	6.57E−51	6	TXNDC12	− 7.0239	1.62E−26
7	GOLGA8VP	6.8771	1.71E−06	7	MFSD14A	− 7.0231	7.74E−07
8	SLC22A14	6.866	2.98E−05	8	GOLGA7	− 6.9895	2.89E−21
9	KCTD5P1	6.8556	3.64E−05	9	TMEM230P2	− 6.9662	2.43E−07
10	KLK14	6.8144	2.52E−06	10	EIF4EP2	− 6.9448	2.15E−07
11	SOCS5P2	6.6905	7.23E−05	11	PSMC1	− 6.8039	4.95E−52
12	XACT	6.6676	0.000663	12	MIR23B	− 6.7505	3.33E−06
13	AQP8	6.5842	0.00015	13	UBE2E1	− 6.6991	1.42E−37
14	PEAK3	6.5725	1.33E−21	14	RAB23	− 6.6506	1.57E−06
15	BEST2	6.5036	0.000181	15	PIGCP1	− 6.5382	2.34E−08
16	PHF24	6.4787	7.98E−07	16	OPN3	− 6.5172	2.15E−08
17	NPW	6.4302	2.70E−54	17	SEC11B	− 6.496	1.08E−05
18	FGF8	6.382	2.89E−05	18	SLC25A5P3	− 6.4548	1.52E−05
19	TUBA3FP	6.3223	0.000393	19	MCM2	− 6.3869	1.84E−66
20	CYB561D2	6.3171	5.21E−07	20	KRT4	− 6.3569	4.13E−08
21	MMP23B	6.3027	7.73E−07	21	PCDHAC2	− 6.3405	3.38E−05
22	OR13J1	6.2333	8.72E−05	22	TUBA1B	− 6.2531	8.30E−161
23	HCRT	6.2212	0.000682	23	LDHAP7	− 6.2398	4.97E−05
24	SBSN	6.2182	9.00E−40	24	ANXA2P1	− 6.1523	1.21E−05
25	EPO	6.1783	0.000732	25	ITGB1P1	− 6.1045	8.85E−50
26	RPS23P6	6.1371	0.001027	26	USP32P1	− 6.0996	9.87E−05
27	NPC1L1	6.1316	1.79E−23	27	SLC4A4	− 6.0966	2.04E−05
28	WFIKKN1	6.0944	8.56E−31	28	DNAJB6P1	− 6.0918	3.83E−07
29	ZGLP1	6.0928	1.50E−52	29	SUMO2P6	− 6.0662	0.000171
30	RPL12P47	6.0862	0.00203	30	DUSP5P1	− 6.0328	0.0001186
179	CLDND2	4.9129	4.23E−83	65	MMP24	− 5.4707	6.3436E−65
318	PINX1	4.4463	1.4025E−75	125	ATP1A1	− 4.8963	6.4343E−75
				199	SHC1	− 4.4469	4.139E−93

Due to the evidence showed above, TCPT might systemically reprogrammed metabolism pattern in SCLC. Mitochondrial is known as the energy generation and supply factory in cells, damaging mitochondrial would highly lead to the change of metabolism patterns. Based on these results and literature evidence, we assumed that TCPT-induced cell death in SCLC may be through metabolic reprogramming by damaging mitochondria.

### β-Catenin/AMPK/SIRT1 axis might be the potential master signalling pathway to reprogram metabolic patterns caused by TCPT in SCLC

**Fig. 3:**
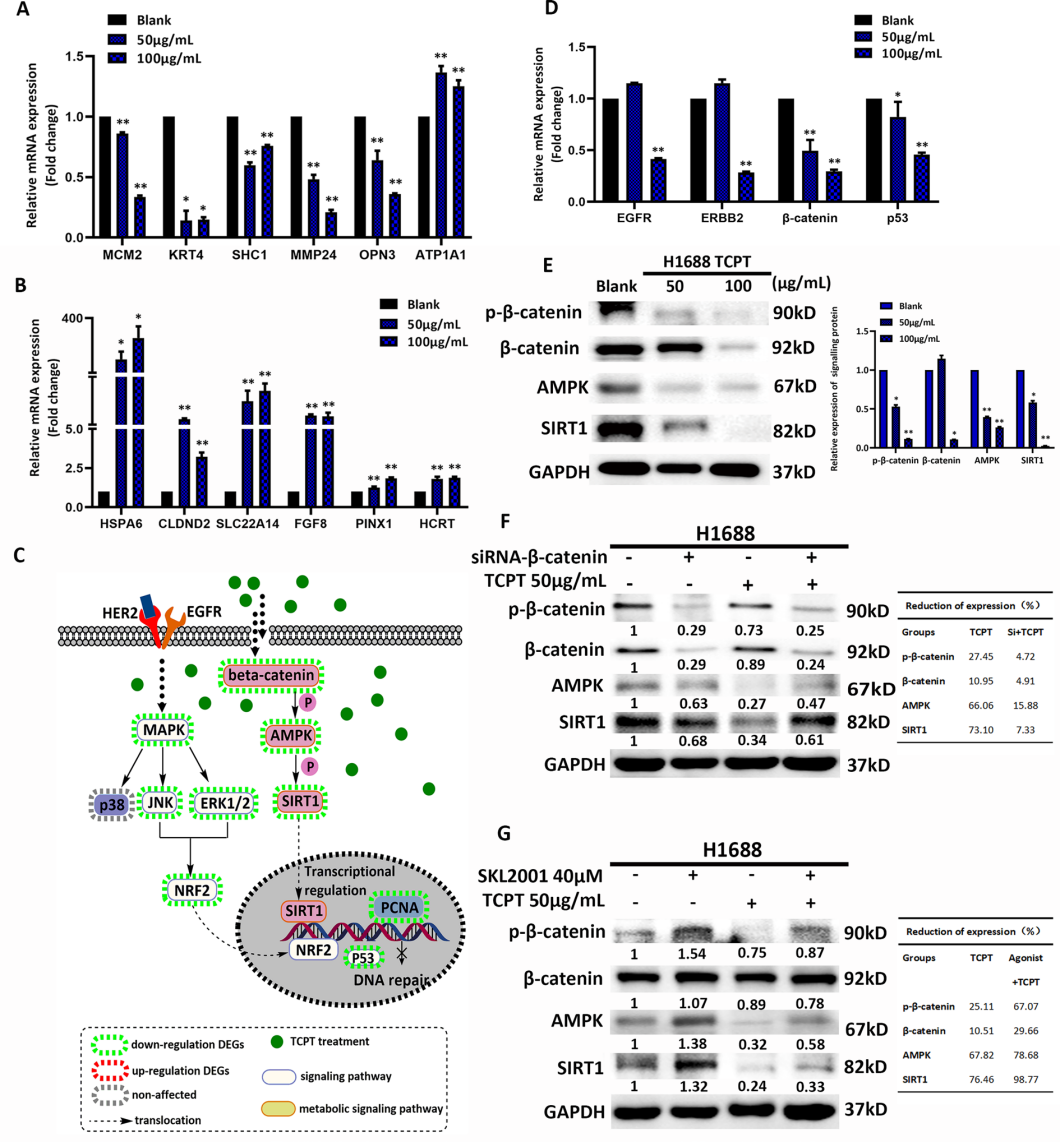
Master signaling pathway potentially regulates metabolism reprogram under TCPT treatment. **A**, **B** Validation of up- and down-regulated DEGs among H1688 blank, 50 μg/mL TCPT and 100 μg/mL TCPT using qPCR. DEGs in each chart are randomly selected from the DEGs list of RNA-seq. **C** Graphic KEGG map of two signaling axis. Details of annotation is labeled in the down part of graph, pink box illustrated the cellular workflow of β-catenin/AMPK/SIRT1 axis. **D** mRNA expression of 4 signaling proteins, EGFR, ERBB2, β-catenin and p53. **E** Protein expression and activation of β-catenin/AMPK/SIRT1 axis. **F**, **G** Rescue assay for detecting whether β-catenin might be the key target when treating TCPT in SCLC. Protein expressions and activation of β-catenin, AMPK and SIRT1 among H1688 with TCPT, combination of TCPT and si-β-catenin, and their counterparts, as well as H1688 with SKL2001 (agonist of β-catenin), combination of TCPT and SKL2001 and their counterparts. Tables in the right panel of **F**, **G** illustrate the relative reduction of expression of each proteins when compared with their no treatment counterparts. *, ** vs blank group, present P value < 0.05 and 0.01 respectively

In this part, we planned to discover which signalling pathways might relate to the metabolic inhibition caused by TCPT. To verify whether the results of RNA-sequencing was accurate, and whether DEGs have the effect of dose-depend manner, the mRNA expression of 6 up-regulated DEGs and 6 down-regulated DEGs were detected. As it was shown in Fig. 3A, B, the expression level of 6 up-regulated DEGs (4 from top 30, 2 from randomly selected among rest) after TCPT treatment were all higher than that of in blank controls. Except ATP1A1 which showed higher mRNA expression in cells with TCPT treatment, the mRNA expression of rest of 5 down-regulated DEGs showed decreased after TCPT treatment compared to their blank counterparts (3 from top 30, 3 from randomly selected among rest). This ensured that the bioinformatics analysis for DEGs identification and enrichment in TCPT-treated cells were dependable. Later, we integrated signalling mapping of KEGG enrichment which involved in potential regulator of metabolism programs together, narrowing the searching ranges. In Fig. 3C, the diagram summarized two pathways, EGFR/MAPK/NRF2 axis and β-catenin/AMPK/SIRT1 axis, related to modulate metabolism patterns in cancers. We first verified 4 metabolic-related DEGs which were also signalling hub among down-regulated list (EGFR, ERBB2, β-catenin, and p53). Figure 3D displayed that after TCPT treatment, the mRNA expression of β-catenin and p53 were decreased in a dose dependent manner in H1688 cells while the drop of mRNA expression of EGFR and ERBB2 were detected when treating with high level of TCPT. In tumor research field, AMPK is well accepted as an important metabolic kinase that regulates ATP replenishing. In normal circumstances, the large consumption of ATP in tumor cells will activate AMPK, thereby activating oxidative phosphorylation or aerobic oxidation to replenish the generation of ATP. Moreover, the protein expression of p-β-catenin, β-catenin, AMPK, and SIRT1 showed decreased in a dose dependent manner in H1688 cells with TCPT treatment (Fig. 3E), compared with their blank counterparts.

Additionally, siRNA was introduced to inhibit the expression of β-catenin in H1688 cells to observe whether the effect of TCPT treatment might be compromised. As it is shown in Fig. 3F, left panel, the expression of p-β-catenin, β-catenin, AMPK, and SIRT1 are down-regulated in H1688 cells that transfected with siRNA-β-catenin (H1688-si). But treating TCPT in H1688-si cells shows slight attenuation of p-β-catenin, AMPK, and SIRT1 expression compared to those of in H1688-si cells (Fig. 3F, right panel). On the other hand, pre-treating H1688 cells with agonist (SKL2001, 40uM, 24 h) significantly up-regulated the activation of β-catenin not the expression of β-catenin in H1688 cells, as well as increased expression of AMPK and SIRT1 (Fig. 3G). Combination SKL2001 and TCPT treatment obviously lowered the expression of p-β-catenin, AMPK, and SIRT1 in H1688 cells compared with that of in single SKL2001 treatment counterparts (Fig. 3G).

These results implied that TCPT treatment had inhibiting effects on metabolic pathway β-catenin/AMPK/SIRT1 axis. Since glycometabolism is the biggest energy source and a main downstream metabolism pattern of β-catenin/AMPK/SIRT1 axis, we concentrated on several glycometabolism-related metabolic patterns which were affected the most by TCPT in the next section.

### TCPT might mainly reprogram aerobic oxidation pattern to reduce ATP production in SCLC

**Fig. 4:**
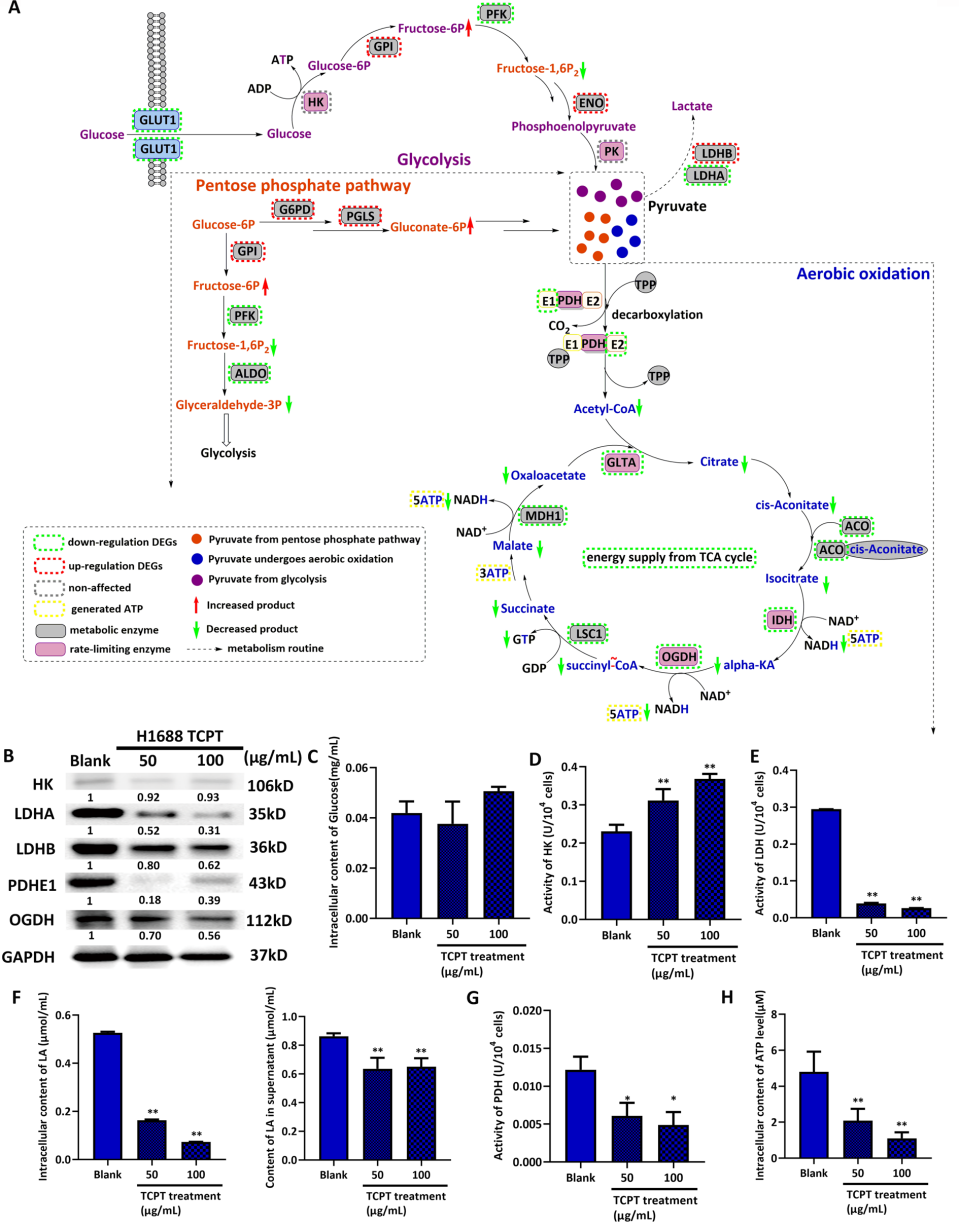
TCPT reprograms metabolic pattern in H1688. **A** Graphic KEGG mapping of 3 ATP-generated metabolism pathway, glycolysis, pentose phosphate pathway and aerobic oxidation. Details annotation are listed in left bottom. Metabolite labels in purple, orange and blue present as intermediate products from glycolysis, pentose phosphate pathway and aerobic oxidation, respectively. According to the results of KEGG mapping, enzymes with green dash line boxes are down-regulated DEGs, red dash line boxes are up-regulated DEGs and grey dash line boxes are non-DEGs. Arrows on green or red indicate the prediction of down- or up-trend of each metabolite. **B** The changes of protein expression of metabolic enzymes in H1688 cell and its TCPT treating counterparts. A series detection of metabolite or activity of key enzymes among H1688 blank, 50 μg/mL TCPT and 100 μg/mL TCPT groups are illustrated in intracellular glucose level (**C**), activity of HK (**D**), activity of LDH (**E**), intracellular and extracellular content of LA (**F**), activity of PDH (**G**) and intracellular content of ATP (**H**). *, ** *vs* blank group, present P value < 0.05 and 0.01 respectively

The metabolically relevant KEGG mappings were selected from all DEGs-involving mapping and were integrated together as illustrated in Fig. 4A. SRIT1 is a key transcriptional factor that mainly regulated the enzymes involving in glycolysis and aerobic oxidation [16]. We found out TCPT showed different regulation towards enzymes in pentose phosphate pathway. But the accumulation of glyceraldehyde-3P in terminal stage of pentose phosphate pathway might be cut down. Drops of glyceraldehyde-3P lead to slowing down ATP formation step in glycolysis (Fig. 4A).

TCPT could also down-regulate the expression of almost all metabolic enzymes in aerobic oxidation, which might lead to the cascade reaction of decreasing ATP production. Glycolysis is the most active metabolic pattern which takes charge of generating multiple intermediates to fuel other metabolism pathways [17]. We found out TCPT treatment showed no effects on the expression of hexokinase (HK) (Fig. 4B), a vital enzyme of the first rate-limiting step in glycolysis. Also, the change of intracellular content of glucose was slightly increased after treating cells with high level of TCPT (Fig. 4C). These results implied that TCPT did not attenuate the major intracellular substrate of glycometabolism. However the activity of HK (Fig. 4D) showed an increased tendency with the raised concentration of TCPT treatment. At this point, the raise of active HK might attribute to the intake of glucose. Moreover, the active HK also meant more consumption of ATP.

Furthermore, the other vital index of glycolysis was formation of lactate (LA) from pyruvate. Since the pyruvate come from several metabolic patterns (Fig. 4A), the activity of lactate dehydrogenase (LDH) and products LA were used for evaluating the activity of glycolysis. As shown in Fig. 4B, E, TCPT treatment lead to the decline of the expression and activity of LDH in a dose dependent manner. The intra- and extracellular content of LA also decreased in a varying extent (Fig. 4F). Formation of LA is the crucial step providing NAD^+^ to respiratory chain for ATP generation. Based on these finding, we preliminary concluded TCPT accelerated ATP consumption but suppressed ATP formation in glycolysis process.

Pyruvate also fuels aerobic oxidation through producing acetylcoenzyme (Acetyl-CoA). In this step, the expression and activity of rate-limiting enzyme pyruvate dehydrogenase E1 (PDHE1) were obviously down-regulated when treating with TCPT in a dose dependent manner (Fig. 4B, G). Besides, TCPT treatment also decreased the expression of oxoglutarate dehydrogenase (OGDH), the third rate-limiting enzyme in tricarboxylic acid cycle (TCA cycle) (Fig. 4B). Finally, the overall intracellular level of ATP content was dramatically lowered in cells with TCPT treatment than that of in blank counterpart (Fig. 4H).

In summary of in vitro results, we theorised TCPT attenuated ATP supply in SCLC cells might attributed to inhibit aerobic oxidation step, partially reduced ATP formation step but stimulated the energy dissipation step in glycolysis cycle.

### In vivo effects of TCPT induced cell death in SCLC through reprogramming metabolic axis β-catenin/AMPK/SIRT1

In in vitro experiments, we revealed that TCPT treatment could induce cell death in SCLC tumor cells maybe because of the damage of mitochondria. The damage of mitochondria could cause metabolic reprogramming such as inhibition of aerobic oxidation step, partial reduction of ATP formation step but stimulation of the energy dissipation step in glycolysis cycle. Overall, metabolism reprogramming caused by TCPT eventually inhibited ATP production. Besides, the metabolic reprogramming might attribute to the suppression of related signalling pathway β-catenin/AMPK/SIRT1 axis. Therefore, in this part we focused on establishing in vivo model to testify the hypothesis based on in vitro results.

**Fig. 5:**
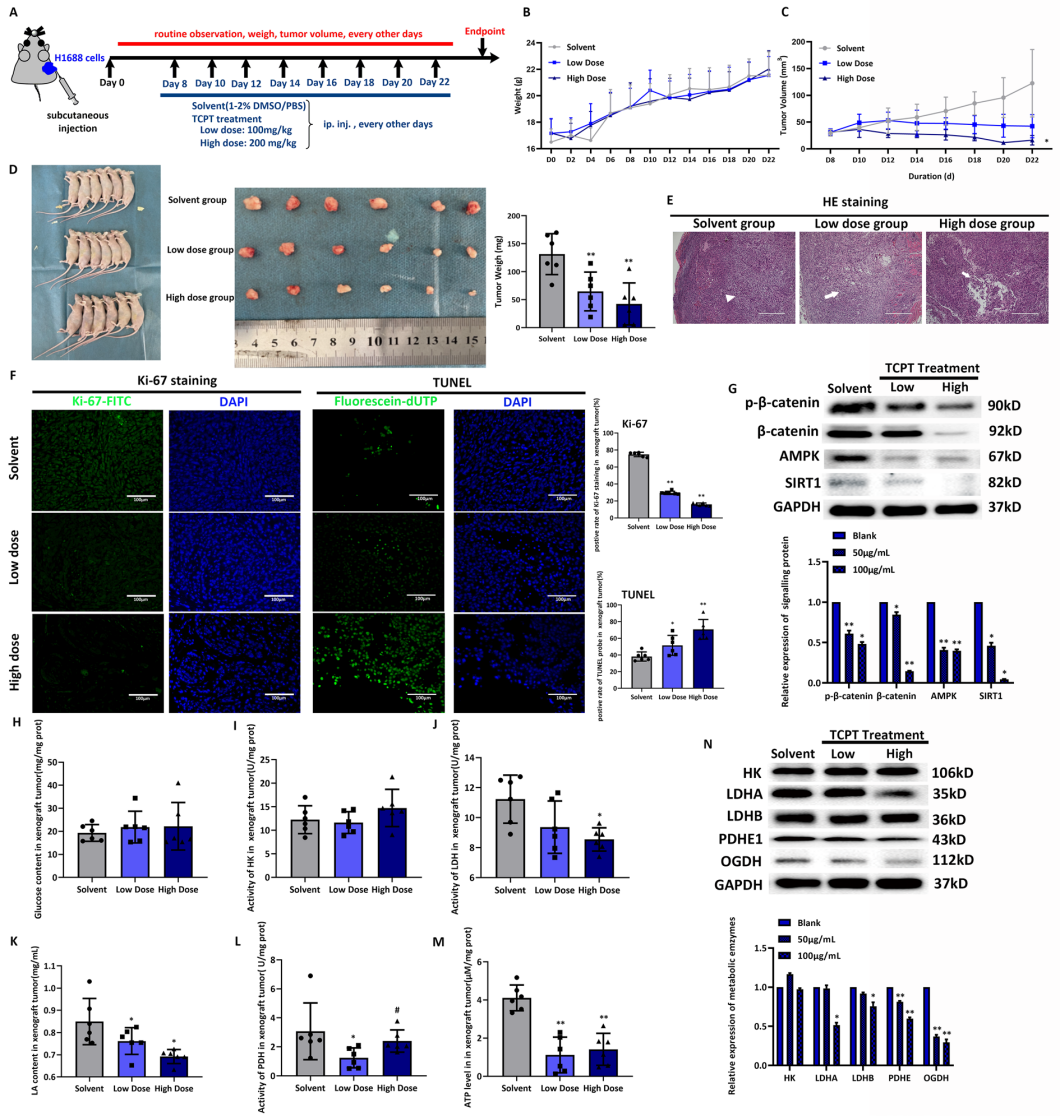
The in vivo mechanism of TCPT in inducing cell death and metabolic reprogramming. **A** The scheme of establishing xenograft model flow and TCPT administration. **B** Record of individual body weight of each group. **C** Record of tumor volume of each group. **D** The sacrificial nude mice, stripped tumors and tumor weight of each group (solvent group, low dose group and high dose group) at experimental endpoint. **E** HE staining display the typical morphological feature in each group. White arrowhead indicates character of SCLC xenograft tumor without TCPT treatment. White arrows illustrate the structure collapse and central necrosis in tumor tissues induced by TCPT treatment. **F** Detection of proliferative index using Ki-67 staining and death index using TUNEL assay among three groups. DAPI staining is used as cell count reference, the positive of Ki-67 or TUNEL staining demonstrates the proliferative activity and death situation respectively in each group. **G** Protein expression of signaling protein among three groups. **H** Glucose content of tumors. I activity of HK in each xenograft tumor among three groups. **J**, **K** The activity of LDH and content of its final products LA in tumor tissue in each group. L The activity of PDH in each tumor tissues. **M** Level of intra-tumorous ATP in each xenograft tissues. **N** the changes of protein expression of rate-limiting enzymes among solvent group and 2 TCPT treating groups. *, ** *vs* blank group, present P value < 0.05 and 0.01 respectively

Figure 5A displayed the process of establishing subcutaneous transplanted tumor model and in vivo TCPT treatment with detailed delivered times and dose. During experimental period, mice weigh and tumor volume were recorded every two days. No obvious change of body weight were investigated among solvent group, low dose group and high dose group (Fig. 5B), indicating the TCPT treatment may not be harmful to nude mice. After 4 days of injection of H1688 cells in mice, Fig. 5C recorded the change of tumor volume after tumor formation till endpoint of experiment, wherein the average tumor volume in low dose group and high dose group stay downhill tendency compared with that of in solvent group. The experiment last 24 days, all mice were sacrificed (Fig. 5D, left panel) and the xenograft tumors were disconnected from mice body (Fig. 5D, middle panel). The stripped xenograft tumors from each group were weighed, the average weighs of xenograft tumor in low dose and high dose were significantly lower than that of in solvent group (Fig. 5D, right panel). HE staining results indicated necrosis lesion appeared (white arrow) at the central area of tumor in mice both in low dose group and high dose group (Fig. 5E). Ki-67 is an indicator of proliferative capacity. Average staining score of Ki-67 in high dose group scored lowest compared with that of in solvent group and low dose group (Fig. 5F, left panel, upper bar chart). The TUNEL staining of Fig. 5F demonstrated the most tumor cells were undergone death progression, showing as high density of positive fluorescein-dUTP staining (Fig. 5F, lower bar chart). These results indicated that TCPT showed in vivo inhibition in tumor growth meanwhile inducing in vivo cell death in SCLC. Besides, the expression of signalling proteins p-β-catenin, β-catenin, AMPK and SIRT1 in each tumor were declined in both low and high dose groups in a different extent, compared to that of in solvent group (Fig. 5G).

Next, we started to detect the changes in metabolic pattern. As it was shown in Fig. 5H, the content of glucose has little changes among xenograft tumor in three groups. Also, the HK activity of xenograft tumor in three groups had no significantly changes either (Fig. 5I). The activity of LDH of in xenograft tumor in low dose group and high dose group were lower than that of in solvent group (Fig. 5J), as well as the content of LA in xenograft tumor shows downtrend in low and high dose group compare to that of in solvent group (Fig. 5K). The average activity of PDH in xenograft tumor in low dose group was significantly lower than that of other two groups (Fig. 5L), which implied high dose of TCPT treatment might induce unexpected slight feedback regulation. Even though some index of metabolic pattern had slightly different from in vitro model, the ATP level in each xenograft tumor in low and high dose groups showed obvious decrease than that of in solvent group (Fig. 5M). Besides, the expression of metabolic protein LDHA, LDHB, PDHB and OGDH were decreased in low and high dose groups in a different extent, compared to that of in solvent group (Fig. 5N). However, the expression of HK was almost the same among three groups (Fig. 5N).

Collectively, these findings demonstrated TCPT induced cell death in SCLC. And this effect might attribute to attenuate β-catenin/AMPK/SIRT1 axis to reprogram metabolic patterns, resulting in decreasing ATP production in vivo and in vitro.

## Discussion

Coumarin derivatives share a basic parental scaffold of α-benzopyrones which consist of benzyl ring condensed lactone structure [18]. Common properties of coumarin derivatives include volatility, solid character with white crystal and vanilla-like smell [19]. Since they widely exist in many plants, extracting from nature medicine is a cost-effective way to obtain large amount of coumarin derivatives. A wide range pharmacological activity in aspects of anti-microbial, anti-oxidant, anti-inflammatory and anticancer activity is found on coumarin derivatives which make them a valuable medicinal material. According to the reports of in vitro studies, coumarin derivatives cause cell cycle arrest in cervical cancer, breast cancer and lung cancer cells, leading to inhibition of proliferation, decrease of genomic replication and decreasing proteins production. Only umbelliferone has been reported as a potential anti-cancer reagent in lung cancer. Treating umbelliferone in lung cancer cells resulted in G1 phase arrest [20] through decreasing expression of cyclin D1 but minimal change in cyclin E and A [21]. Besides, exposing lung cancer cells in umbelliferone also lead to activation of caspase-3, decreased expression of BCL-2 and induced cell apoptosis [22, 23]. Moreover, analogue of umbelliferone dramatically lowered effective concentration and brought mitochondrial damage in lung cancer cells [24]. These reports imply umbelliferone is a potential lead compound with good anticancer activity. The rest of two component 7-hydroxy-8-methoxycoumarin and skimmin have not yet been reported with good anti-cancer activity. However, skimmin has been reported as a renal protective reagent [25]. This evidence implies TCPT might contain both cytotoxicity to cancer cells and protection to normal cells. In accordance with our results, no symptom of systemic dyscrasia was observed in TCPT treatment group in mice model. The In vitro model showed that low dose of TCPT stimulated proliferation of normal lung cells, despite inhibition rate of normal cells is relatively similar to that of lung cancer cells at high dose. In vivo model also showed little toxicity of TCPT since no significant reduction of weight and visceral index were found in nude mice with TCPT treatment (Additional file 4: Table S4). Taken together, our results suggested TCPT extract from *Pileostegia tomentella* Hand. Mazz is an effective and hypotoxic anti-cancer reagent for SCLC cells.

Additionally, current literatures indicated coumarin derivatives induced apoptosis of lung cancer cells through mitochondrial dependent pathway or terminal apoptotic signal, activation of caspase-3 pathway [26]. This might attribute to the damage of mitochondrial function by nucleophile *cis*-o-hydroxy cinnamate which generated from ring cleavage of lactone [27]. Recently, various programmed cell death has been sub-classified as ferroptosis, pyropotosis, cuproptosis and necroptosis due to the different lead inducement. As illustrated in the result of FCM-apoptosis detection, TCPT treatment increased numbers of cells with positive staining of nuclear and double positive staining of nuclear/Annexin-V. Since positive staining of nuclear also one of the symbol of ferroptosis, pyropotosis and necroptosis, this result indicated besides apoptosis, different types of cell death might be co-existed in SCLC under the TCPT inducement. In addition, ultrastructure changes observed by TEM also found out the early occurrence of autophagolysosome derived from mitochondria, as well as autophagic vesicles of unknown origin and suspected pyropototic body. Researches also provided evidence that backbone of umbelliferone was necessary for inducing oxidative stress through inserting Kelch1-6 domain of Keap1 protein [28]. The activation of Keap1 leads to intracellular iron accumulation which causes ferroptosis in cancer cells. Our previous reports found out TCPT activated inflammation in H_22_ tumor bearing mice through producing IL-2 and TNF-α [11], which imply TCPT might be a pro-inflammatory factor in cancer treatment. Despite pyropotosis and necroptosis are both inflammation-orientated cell death, being responsible of pyropotosis or necroptosis of TCPT in SCLC through inducing pro-inflammatory still need further experimental validation.

Based on our current results, the most up-regulated DEGs, HSPA6, might be the potential suppressive targets of TCPT. Latest researches imply that the up-regulation of HSPA6 relates to drug response in cancer therapy. Sekino et al. reported that high level of HSPA6 induced by metformin treatment predicted a better prognosis in patients with stage I/II esophageal squamous cell carcinoma [29]. Shen et al. found out that increased expression of HSPA6 predicted a longer overall survival in patients with triple-negative breast cancer (TNBC) and accepted thymoquinone treatment. The in vitro model also indicated high HSPA6 expression inhibited the proliferation, migration and invasion of TNBC cell lines [30]. Besides, treating lung cancer cells with Manumycin A induced up-regulation of HSPA6, which could form a positive feedback loop and sensitize cancer cells to Manumycin A treatment [31]. On the contrast, one of the down-regulated DEGs OPN3 (ranks 16 of most down-regulated list) might be the potential oncogene target. High expression of OPN3 in tumor tissue associated with poor prognosis and cancer-associated fibroblast infiltration [32]. Additionally, melanoma patients carrying higher expression of OPN3 have shorter overall survival and occurrence of ulceration [33]. So far no researches have reported the biological function of neither HSPA6 nor OPN3 in SCLC. Based on the literature reviews and our results, the up-regulated HSPA6 may be the protection of stress-respond in SCLC when treating with TCPT.

Another research prospect is TCPT treatment potentially affects pro-drug chemo-reagents of ‘first-line’-used, such as irinotecan, capecitabin and cisplatin in SCLC. Additional file 5: Fig. S1 integrated KEGG mapping result of DEGs that involved in drug metabolism pathways. Two major ‘drug pump’, P-gp and MRP2 showed decreased expression, which implies TCPT might stably maintain the cellular concentration of chemo-reagents. Besides, declined expression of drug-metabolizing enzymes CYP3A4 and CES1 also delay intracellular inactive transformation of those chemo-reagents. On the contrary, accelerated active transformation of those chemo-reagents might due to the up-regulated expression of CYP2A6 and TYMP. Taken together, combination of TCT with first-line chemo-reagents might potentially increase their chemo-sensitization. The damage of mitochondria caused by TCPT affects the expression of BCL-2 very little but induces higher expression of apoptotic agonists BAX and BAD. Besides, the expression of typical mitochondrial-apoptotic protein cytochrome C has decreased which accordance with our results in Fig. 1G. Surprisingly, the reduced expression of caspase family in down-stream sites of apoptotic pathways might lead to cell death as other pathway instead of classical mitochondrial-related apoptosis. These results imply that TCPT treatment induces cell death maybe through activating pan-death pathways, wherein multiple types of cell death might be co-existed together.

Under physiological conditions, most of energy supplement comes from high efficacy metabolism, TCA cycle. On the other hand, tumor cells prefer glycolysis to supply energy, a low efficacy patterns but generate more intermediates for boosting other metabolism pathways. Specifically interpreting, current researches revealed that SCLC leaned to initiate amino acid metabolism supplement such as glutamine or arginine metabolism so that bypassing glycolysis to reduce energy self-consuming. In this study, both in vitro and in vivo data indicated TCPT treatment significantly reduced the ATP level in SCLC without affecting intracellular glucose level. In details, TCPT might induce accumulation of pyruvate due to increasing HK activity and decreasing activity and expression of LDHA and LDHB. These results imply that the primary material of TCA cycle from glycolysis is sufficient, which means the attenuation of ATP production might not be lack of pyruvate. Next, we found that the expression and activity of PDHE were also down-regulated with the increasing of TCPT concentration, thereby declining production of acetyl-CoA. Many reports displayed that SCLC cells also motivate glutamine and pentose phosphate pathways to replenish α-ketoglutarate (α-KA). Additionally, α-KA needs to be transformed to succinyl-CoA by OGDH thereby acquiring 5 equivalent amount of ATP. However, TCPT treatment also cut down α-KA-orientated ATP production by declining the expression of OGDH in in vitro and in vivo model. Taken together, TCPT down-regulated the expression or activity of rate-limiting enzymes and decreased ATP production in SCLC. Draining energy supplement has been accepted as a sufficient therapeutic strategy for inducing cell death in SCLC. Latest research demonstrates inhibiting glycolysis rate-limiting enzyme 6-phosphofructo-2-kinase (PFK2) effectively attenuate malignant phenotype including growth ability, stemness and metastasis potential in SCLC [34]. Lowering activity of HK suppressed proliferation, invasion and migration in SCLC [35]. On the other hand, elevated LDH level and HK2 expression in SCLC predicts higher malignant circumstance and worse prognosis [36, 37].

Different driven-phenotype of SCLC cells alternate specific preponderant metabolism patterns to fuel biology progression. SCLC cells are basically classified as neuroendocrine type, ASCL1, NEUROD1, POU2F3 and YAP1 and non- neuroendocrine, with low MYC expression subtypes [38]. For instance, SCLC cells carrying low ASCL1 expression have more active pattern of de novo guanosine nucleotide synthesis [39]. Moreover, SCLC cells without MYC over-expression perfected launching aerobic oxidation pattern while glutamine metabolism and glycolysis are priorities metabolism patterns in MYC-addicted SCLC cells [40]. H1688 cell line obtains over-expression of wild type TP53 and moderate expression of MYC, so that it is more similar to non-neuroendocrine subtype. This phenotype characteristics indicated that predominant metabolic pattern was highly possible the aerobic oxidation in H1688, and it might be also explained why TCPT treatment affected almost all enzymes in the progression of aerobic oxidation in H1688 cells.

Indeed, this study could provide more information once few limitations are overcome. For examples, whether TCPT reprogrammed other metabolism patterns or similar pattern in neuroendocrine type SCLC. Whether TCPT treatment induces the similar metabolism alternation in the other cancer with the same addicted-phenotype also remains further discovered. Those questions are worthy to further discuss in the future. Besides, modification of in vivo model might also provide more evidence in terms of immune regulation of TCPT treatment. In current study, immune deficient mice such as SCID mice and nude mice are prevalently applied to tumor research. In traditional Chinese medicine, the etiology of tumors is mostly attributed to stasis of phlegm and dampness. Tumor therapy in Chinese medicine mainly aims at systemic recuperation such as strengthening the body resistance to eliminate pathogenic factors, balancing yin-yang harmony and activating immune defection. However, mild immune respond in immune deficient mice has been a challenge to fully mimic the in vivo progression in human body. Recently, zebrafish which is vulnerable to xenograft tumor cells and acquires full immune respond is introduced as a high effective and a more emulational model in tumor in vivo study [41, 42]. We may observe much better anti-cancer effect under the coordination of immune regulation in in vivo model with intact immune system. Applying these modifications may let us understand the further pharmacological mechanism of action of TCPT better and also bring new thoughts in modification of chemical structure and pharmaceutical preparation.

## Conclusion

In this study, we establish in vitro and in vivo model to find out the anti-tumor effect of TCPT in SCLC. The underlying mechanism is that TCPT reprograms metabolic patterns to reduce ATP production, possibly through attenuating master metabolic pathway axis β-catenin/AMPK/SIRT1. It is the first time illustrating that the mixture of coumarins derivative from Yao medicine exhibits good anti-tumor effect in SCLC through metabolic reprogramming. In order to move deeper to more preclinical progress, further study could focus on the structural modification to yield much better anti-tumor activity, or capsuling mixture into specific delivery systems to reach tumor accurately.

## Supplementary Information

**Additional file 1: Table S1. **List of antibodies and chemicals.

**Additional file 2: Table S2. **Lung cancer cell lines and their culture condition.

**Additional file 3: Table S3. **The list of primers involved in this study.

**Additional file 4: Table S4. **List of index of organs in each group.

**Additional file 5: Figure S1. **Drug metabolism pathway and apoptosis pathway of DEGs.

## Data Availability

All data and related information were included in this published paper, which could be acquired in the figures, tables and supplemental materials. Data of this study is available by the authors, without undue reservation.

## Abbreviations

OD: Optical Density
HSPA6: Heat shock protein family A (Hsp70) member 6
OPN3: Opsin 3
MRP2: ATP binding cassette subfamily C member 2
P-gp: ATP binding cassette subfamily B member 1
CYP3A4: Cytochrome P450 family 3 subfamily A member 4
CES1: Carboxylesterase 1
CYP2A6: Cytochrome P450 family 2 subfamily A member 6
TYMP: Thymidine Phosphorylase
BCL2: BCL2 apoptosis regulator
BAX: BCL2 associated X, apoptosis regulator
BAD: BCL2 associated agonist of cell death
ASCL1: Achaete-scute family bHLH transcription factor 1
NEUROD1: Neuronal Differentiation 1
POU2F3: POU class 2 homeobox 3
YAP1: Yes1 associated transcriptional regulator
MYC: MYC proto-oncogene, bHLH transcription factor
TP53: Tumor protein p53
SCID: Severe combined immune deficiency
