# Supplementary material for: Total coumarins of Pileostegia tomentella induces cell death in SCLC by reprogramming metabolic patterns, possibly through attenuating β-catenin/AMPK/SIRT1
Source: Chin Med. 2023 Jan 3;18:1. doi: 10.1186/s13020-022-00703-7 (PMC9809065; doi:10.1186/s13020-022-00703-7)
Supplement: Supplementary file 1 — Additional file 1: Table S1. List of antibodies and chemicals. [file 13020_2022_703_MOESM1_ESM.doc]

**Table 1S List of Antibodies and Chemicals**

| **Chemicals** | | | | | | |
| --- | --- | --- | --- | --- | --- | --- |
| **Name** | | **CAS** | | | **suppliers** | |
| 7-hydroxycoumarin | | 93-35-6 | | | Aladdin(H109352) | |
| skimmin | | 93-39-0 | | | Macklin(S818260) | |
| SKL2001 | | 909089-13-0 | | | MCE(HY101085) | |
| 7-hydroxy-8-methoxycoumarin | | 485-90-5 | | | Macklin(H878860) | |
| CCK-8 (WST) | | 193149-74-5 | | | DOJINDO(CK04) | |
| DAPI | | 28718-90-3 | | | Thermo Fischer(R37606) | |
| Lipo2000 | | NA | | | Thermo Fischer(11668500) | |
| **Antibodies** | | | | | | |
| **Name** | **species** | | **suppliers** | **LOT** | | **diluted** |
| GAPDH | Rabbit | | BBI | D110016-0100 | | 1:2000 |
| p-β-catenin | Rabbit | | Proteintech | 23000093 | | 1:5000 |
| β-catenin | Rabbit | | Proteintech | 00105595 | | 1:5000 |
| AMPK | Rabbit | | Proteintech | 00099549 | | 1:2000 |
| SIRT1 | Rabbit | | Proteintech | 00102771 | | 1:2000 |
| Caspase 3 | Mouse | | CST | 9668S03 | | 1:1000 |
| Cytochrome C | Mouse | | Proteintech | 10008353 | | 1:5000 |
| OGDH | Rabbit | | Proteintech | 00095430 | | 1:5000 |
| HK | Rabbit | | Proteintech | 00085617 | | 1:2000 |
| PDHE1 | Rabbit | | Proteintech | 00101770 | | 1:2000 |
| LDHA | Rabbit | | Proteintech | 00051270 | | 1:5000 |
| LDHB | Rabbit | | Proteintech | 00105268 | | 1:5000 |
| Ki-67 | Mouse | | ZSCB-BIO | 22051754 | | 1:100 |
| Alex 488-goat anti Ms IgG | Goat | | Shangon | D11061-0100 | | 1:100 |

*****
