# Supplementary material for: Total coumarins of Pileostegia tomentella induces cell death in SCLC by reprogramming metabolic patterns, possibly through attenuating β-catenin/AMPK/SIRT1
Source: Chin Med. 2023 Jan 3;18:1. doi: 10.1186/s13020-022-00703-7 (PMC9809065; doi:10.1186/s13020-022-00703-7)
Supplement: Supplementary file 2 — Additional file 2: Table S2. Lung cancer cell lines and their culture condition. [file 13020_2022_703_MOESM2_ESM.doc]

**Table S2 Lung cancer cell lines and their culture condition**

| **Name** | **Subtype** | **RRID** | **Basic medium** | **supplement** |
| --- | --- | --- | --- | --- |
| BEAS-2B | normal | CVCL_0168 | BEBM | Penicillin-Streptomycin(100U), BPE(0.4%), 0.1% hydrocortisone, hEGF(0.1%), epinephrine(0.1%), insulin(0.1%), transferrin(0.1%), triiodothyronine(0.1%), retinoic acid(0.1%) |
| NCI-H1688 | SCLC | CVCL_1487 | RMPI-1640 | 10%FBS, Penicillin-Streptomycin(100U), L-glutamine(2mM), Na_2_CO_3_(1.5g/L) |
| NCI-H446 | SCLC | CVCL_1562 | RMPI-1640 | 10%FBS, Penicillin-Streptomycin(100U), L-glutamine(2mM), Na_2_CO_3_(1.5g/L) |
| A549 | LUAD | CVCL_IY87 | RMPI-1640 | 10%FBS, Penicillin-Streptomycin(100U), L-glutamine(2mM), Na_2_CO_3_(1.5g/L) |
| NCI-H1299 | LUAD | CVCL_0060 | RMPI-1640 | 10%FBS, Penicillin-Streptomycin(100U), L-glutamine(2mM), Na_2_CO_3_(1.5g/L) |
| NCI-H226 | LUSC | CVCL_1544 | RMPI-1640 | 15%FBS, Penicillin-Streptomycin(100U), L-glutamine(2mM), Na_2_CO_3_(1.5g/L) |
| SK-MES-1 | LUSC | CVCL_0630 | EMEM | 10%FBS, Penicillin-Streptomycin(100U), L-glutamine(2mM), sodium pyruvate(1mM), Na_2_CO_3_(1.5g/L) |

*SCLC: small cell lung cancer. LUAD: lung adenocarcinoma; LUSC: squamous carcinoma; FBS: fetal bovine serum; BPE: Bovine Pituitary Extract; hEGF: human Epidermal Growth Factors
