# Supplementary material for: Total coumarins of Pileostegia tomentella induces cell death in SCLC by reprogramming metabolic patterns, possibly through attenuating β-catenin/AMPK/SIRT1
Source: Chin Med. 2023 Jan 3;18:1. doi: 10.1186/s13020-022-00703-7 (PMC9809065; doi:10.1186/s13020-022-00703-7)
Supplement: Supplementary file 3 — Additional file 3: Table S3. The list of primers involved in this study. [file 13020_2022_703_MOESM3_ESM.doc]

Table S3. The list of primers involved in this study

| **Primers** | **Forward 5’→3’** | **Reverse 5’→3’** |
| --- | --- | --- |
| MCM2 | GGCGAGGAGGACGAGGAGATG | AAGTTCTTGAAGCGGTGGTGGATC |
| KRT4 | AACGCACAGCAGCCGAGAATG | GCCTCCAACTCCACCTTGTTCAG |
| SHC1 | ATCACTCTCACCGTCTCCACCAG | TTCACAGGGTCTTTGGCAACATAGG |
| MMP24 | CACTCACCATCGGAGAGGAAACAC | GTTGAAGTTGCCGTCACAGATGTTG |
| OPN3 | TGCTGGTGCTCGTCCTCTACTAC | CAGGTCGCTGAGGCTGATGTTG |
| ATP1A1 | GGATGACCGCTGGATCAACGATG | CACCACCACGATACTGACGAAGAAG |
| HSPA6 | CTAATGGCATCCTGAGCGTGACAG | CCATCCTCTCCACCTCCTCCTTG |
| CLDND2 | CTGCTGCTGACCGCCTTGATAG | GACACACGGGGAATCCACTGATG |
| SLC22A14 | CTGTTTCTGGTGGGTGGGATACTTG | TCTTGTTCACACTTGCGGCGTAG |
| FGF8 | CGGACACCTTTGGAAGCAGAGTTC | TGCCTTTGCCGTTGCTCTTGG |
| PINX1 | ACACTGCCTGGAGTAATGACGATTC | TCTGTGGCTCCTTGCTCCTGAG |
| HCRT | CTACTGCTGCTGCTGCTGCTG | AGCTCGTAGAGGCGGCAAGAG |
| EGFR | TCCAACTTCTACCGTGCCCT | CCCTGCTGTGGGATGAGGTA |
| ERBB2 | GTACACGATGCGGAGACTGC | GCACCTTCACCTTCCTCAGC |
| β-catenin | TGTGACTTGCACGTACTCCC | | ACCATCGCTATCTGAGCAGC | | --- | |
| p53 | CTGATCCTTCATATGAAGCAGCA | CCTGAAACAAACTTTCATCGGTG |
| si β-catenin | GAUGGUGUCUGCUAUUGUACGTT | CGUACAAUAGCAGACACCAUCTT |
