# Supplementary material for: Total coumarins of Pileostegia tomentella induces cell death in SCLC by reprogramming metabolic patterns, possibly through attenuating β-catenin/AMPK/SIRT1
Source: Chin Med. 2023 Jan 3;18:1. doi: 10.1186/s13020-022-00703-7 (PMC9809065; doi:10.1186/s13020-022-00703-7)
Supplement: Supplementary file 4 — Additional file 4: Table S4. List of index of organs in each group. [file 13020_2022_703_MOESM4_ESM.docx]

| Group | Average Index of organs (n=6) | | | | |
| --- | --- | --- | --- | --- | --- |
|  | **Heart** | **Liver** | **Spleen** | **Lung** | **Kidney** |
| Solvent | 0.607±0.018 | 6.198±0.476 | 0.566±0.048 | 0.631±0.041 | 0.809±0.099 |
| Low dose | 0.572±0.040 | 6.243±0.461 | 0.569±0.093 | 0.639±0.036 | 0.786±0.076 |
| High dose | 0.578±0.020 | 6.310±0.328 | 0.502±0.110 | 0.668±0.024 | 0.831±0.040 |
| P value | - | - | - | - | - |

**Table S4 List of index of organs in each group**

*Index of organs follows the equation: organ weight (g)/body weight (g)×100
