# Supplementary figures and images for: Total coumarins of Pileostegia tomentella induces cell death in SCLC by reprogramming metabolic patterns, possibly through attenuating β-catenin/AMPK/SIRT1
Source: Chin Med. 2023 Jan 3;18:1. doi: 10.1186/s13020-022-00703-7 (PMC9809065; doi:10.1186/s13020-022-00703-7)

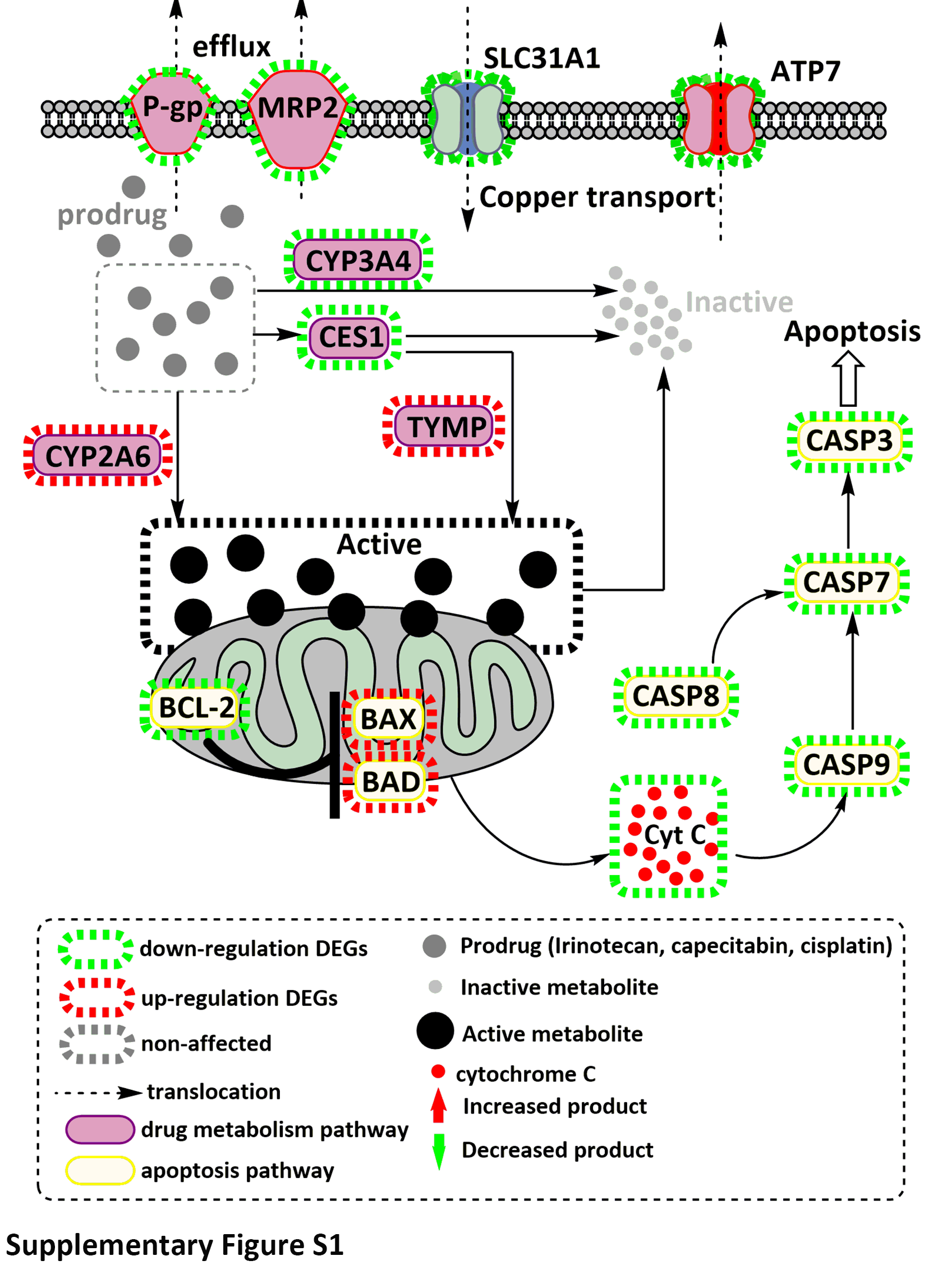

Supplement: Supplementary file 5 — Additional file 5: Figure S1. Drug metabolism pathway and apoptosis pathway of DEGs. [file 13020_2022_703_MOESM5_ESM.tif]
